# Real-world effectiveness of third- or later-line treatment in Japanese patients with HER2-positive, unresectable, recurrent or metastatic gastric cancer: a retrospective observational study

**DOI:** 10.1007/s10147-022-02162-4

**Published:** 2022-04-30

**Authors:** Daisuke Sakai, Takeshi Omori, Soichi Fumita, Junya Fujita, Ryohei Kawabata, Jin Matsuyama, Hisateru Yasui, Motohiro Hirao, Tomono Kawase, Kentaro Kishi, Yoshiki Taniguchi, Yasuhiro Miyazaki, Junji Kawada, Hironaga Satake, Tomoko Miura, Akimitsu Miyake, Yukinori Kurokawa, Makoto Yamasaki, Tomomi Yamada, Taroh Satoh, Hidetoshi Eguchi, Yuichiro Doki

**Affiliations:** 1 Osaka University Graduate School of Medicine, Suita, Japan; 2 Osaka International Cancer Institute, Osaka, Japan; 3 Kindai University, Osakasayama, Japan; 4 Sakai City Medical Center, Sakai, Japan; 5 Osaka Rosai Hospital, Sakai, Japan; 6 Higashiosaka City Medical Center, Higashiosaka, Japan; 7 Kobe City Medical Center General Hospital, Kobe, Japan; 8 National Hospital Organization Osaka National Hospital, Osaka, Japan; 9 Toyonaka Municipal Hospital, Toyonaka, Japan; 10 Osaka Police Hospital, Osaka, Japan; 11 Saiseikai Senri Hospital, Suita, Japan; 12 Osaka General Medical Center, Osaka, Japan; 13 Yao Municipal Hospital, Yao, Japan; 14 Kansai Medical University, Hirakata, Japan; 15 Daiichi Sankyo Co., Ltd., Tokyo, Japan; 16 Osaka University Hospital, Suita, Japan; 17 Present Address: Yao Municipal Hospital, Yao, Japan; 18 Present Address: Sakai City Medical Center, Sakai, Japan; 19 Present Address: Tohoku University School of Medicine, Sendai, Japan; 20 Present Address: Kansai Medical University, Hirakata, Japan

**Keywords:** Gastric cancer, HER2 +, Third- or later-line treatment, Nivolumab, Japan

## Abstract

**Background:**

Real-world evidence on the preference for and effectiveness of third- or later-line (3L +) monotherapy for HER2-positive gastric cancer is limited in Japan. This study evaluated the utility of nivolumab, irinotecan, and trifluridine/tipiracil (FTD/TPI) monotherapy as 3L + treatment in Japanese patients with HER2-positive gastric/gastroesophageal junction (G/GEJ) cancer who were previously treated with trastuzumab.

**Methods:**

In this multicenter, retrospective, observational study (20 centers), data of eligible patients were extracted from medical records (September 22, 2017–March 31, 2020), with follow-up until June 30, 2020. Outcomes included overall survival (OS), real-world progression-free survival (rwPFS), time to treatment failure (TTF), objective response rate (ORR; complete response [CR] + partial response [PR]), and disease control rate (DCR).

**Results:**

Of 127 enrolled patients, the overall analysis population comprised 117 patients (median [range] age, 71 [38–89] years). The most commonly prescribed 3L + monotherapy was nivolumab (*n* = 100), followed by irinotecan (*n* = 12) and FTD/TPI (*n* = 5). The median (95% confidence interval [CI]) OS, rwPFS, and TTF were 6.2 (4.5–8.0), 1.9 (1.5–2.3), and 1.8 (1.5–2.2) months, respectively, at median (range) 150 (25–1007) days of follow-up. The ORR (CR + PR) and DCR were 9.0% (1% + 8%) and 32.0%, respectively. Factors such as higher neutrophil–lymphocyte ratio (≥ 2.54), Glasgow prognostic score (≥ 1), Eastern Cooperative Oncology Group performance status (ECOG PS; ≥ 2), and hepatic metastasis significantly impacted OS.

**Conclusions:**

The observed OS in this study for HER2-positive G/GEJ cancer was shorter than that reported previously, suggesting that the effectiveness of nivolumab, irinotecan, or FTD/TPI as 3L + therapy may be limited.

**Supplementary Information:**

The online version contains supplementary material available at 10.1007/s10147-022-02162-4.

## Introduction

Based on the GLOBOCAN 2018 estimates, gastric cancer is among the five most common newly diagnosed cancers as well as among the top five leading causes of cancer-related deaths worldwide [1]. In 2018, gastric cancer accounted for 782,685 new deaths globally and 1,033,701 new cases of gastric cancer were reported worldwide [1]. In Japan, gastric cancer was the second most common newly diagnosed cancer after colon cancer in 2018 and the third most common cause of cancer-related deaths in 2019 [2] despite a significant decrease in its incidence and mortality in the past 3 decades [3].

The frequency of human epidermal growth factor receptor 2 (HER2)–expressing gastric/gastroesophageal junction (G/GEJ) cancer was comparable between patients evaluated for targeted therapy in Japan (21.2%) versus worldwide, including Europe and the United States (20–22.1%) [4–6]. However, treatment options specific to HER2-positive, unresectable, recurrent or metastatic G/GEJ cancer are limited. The Japanese Gastric Cancer Association (JGCA) guidelines, revised in January 2018, recommend capecitabine (or S-1) and cisplatin plus trastuzumab as standard first-line therapy for HER2-positive G/GEJ cancer [7], primarily based on the results of the Trastuzumab for Gastric Cancer (ToGA) study [6]. A combination of ramucirumab and paclitaxel is recommended as second-line therapy based on the results of the RAINBOW trial as there is no anti-HER2-specific therapy for patients who have failed treatment with trastuzumab [8]. Nivolumab, irinotecan, or trifluridine/tipiracil (FTD/TPI) monotherapy is recommended as third-line treatment, irrespective of HER2 status [7, 9].

The efficacy of the currently recommended third- or later-line treatment for gastric cancer has been demonstrated [10–13]. In the ATTRACTION-2 trial, nivolumab, an anti–programmed death-1 (PD-1) antibody, showed a survival benefit over placebo (median overall survival [OS] duration: 5.26 vs 4.14 months; median progression-free survival [PFS] duration: 1.61 vs 1.45 months) in patients who progressed after standard therapy [10]. Newly initiated irinotecan monotherapy as third- or later-line chemotherapy has demonstrated a median OS of 4.0–6.6 months [11, 12]. The TAGS trial demonstrated a survival benefit of FTD/TPI monotherapy over placebo (median OS: 5.7 vs 3.6 months) [13].

However, real-world evidence on the effectiveness of third- or later-line monotherapy for HER2-positive gastric cancer is limited in Japan. It is also important to understand physicians’ preferences and the efficacy of third- or later-line treatment to explore future treatment strategies.

The objective of this study was to clarify the utility of nivolumab, irinotecan, and FTD/TPI monotherapy as third- or later-line treatment in Japanese patients with HER2-positive G/GEJ cancer who have previously received trastuzumab treatment.

## Methods

### Study design

This multicenter, retrospective, observational study was conducted at 20 centers in Japan. Data of Japanese patients aged ≥ 20 years with HER2-positive, unresectable, or recurrent gastric cancer who were newly initiated on nivolumab, irinotecan, or FTD/TPI monotherapy as third- or later-line therapy and had been previously treated with trastuzumab were extracted from medical records between September 22, 2017, and March 31, 2020. Perioperative chemotherapy was counted as first-line treatment in patients who had received perioperative chemotherapy and had recurrence during or within 6 months after postoperative chemotherapy or noncurative resection. Eligible patients were followed up until June 30, 2020 (data cutoff date), to enable a minimum of 3 months of observation. Data were extracted by the principal investigator using the medical record retrieval systems of the participating institutions. Thereafter, the principal investigator assigned patient identification codes to all patients who met the inclusion criteria and did not violate the exclusion criteria. The identification codes were recorded along with patient-identifiable information and stored at the participating institution. All patients were registered using the case information collection system DATATRAK ONE^®^ (DATATRAK International, Inc, Mayfield Heights, OH, USA). Patient information was entered into DATATRAK ONE^®^ from the medical records of all patients who were assigned an identification code without including personally identifiable information. Nivolumab was approved by the Japanese Ministry of Health, Labour and Welfare (MHLW) on September 22, 2017, for patients with unresectable advanced or recurrent gastric cancer who had progressed after chemotherapy [14]. HER2-positive G/GEJ cancer was pathologically diagnosed using the updated gastric cancer handling convention (immunohistochemistry [IHC] or in situ hybridization [ISH]) by the attending physician (IHC3 + or IHC2 + /ISH + was considered HER2 positive) [15].

This study was conducted in accordance with the Declaration of Helsinki and the Ethical Guidelines for Medical and Health Research Involving Human Subjects. The study protocol was reviewed and approved by the ethics review committee of each participating medical institution. Informed consent was not applicable to this study, and an opt-out approach was adopted to ensure an opportunity for participants to refuse participation in the study. Patients who declined to participate in the study before data fixation were excluded. The UMIN-CTR registration identifier of the study is UMIN000040853 (https://upload.umin.ac.jp/cgi-open-bin/ctr_e/ctr_view.cgi?recptno=R000046480).

### Outcomes

The treatment outcomes of interest were OS, real-world PFS (rwPFS), duration of response (DOR), time to treatment failure (TTF), objective response rate (ORR; complete response [CR]/partial response [PR]), and disease control rate (DCR). Tumor response was evaluated per the Response Evaluation Criteria in Solid Tumours (RECIST) version 1.1 in principle, but evaluation by investigators at the time of treatment was prioritized. In addition, progressive disease (PD) may have been judged not only by imaging (such as a computed tomography [CT] scan) but also as clinical PD by the investigators. OS was defined as the period from the start date of the relevant treatment/monotherapy until death from any cause. rwPFS was defined as the period from the start date of treatment/monotherapy to disease progression or death from any cause, whichever occurred first. DOR was defined as the period from the date on which tumor response (CR or PR) was confirmed to the date on which PD was confirmed for the first time or the date of death from any cause. DOR was assessed only in patients with tumor response (CR or PR). TTF was defined as the period from the date of initiation of treatment/monotherapy to disease progression, early discontinuation of treatment, or death from any cause, whichever occurred first.

An exploratory analysis was also performed to evaluate the OS, rwPFS, TTF, and ORR in the overall population and the nivolumab subgroup of this study, similarly matched to the DESTINY-Gastric01 (DG01, NCT03329690) partial inclusion criteria, evaluable lesions, and Eastern Cooperative Oncology Group performance status (ECOG PS) 0 or 1 [16]. Hereafter, we call this analysis the “DG01 similarly matched analysis.” The phase 2 DG01 study evaluated trastuzumab deruxtecan (T-DXd) versus chemotherapy (physician’s choice [PC], irinotecan or paclitaxel) in patients with HER2-positive advanced gastric cancer [16]. As nivolumab was not included in the PC group in the DG01 study, the purpose of this analysis was to estimate the efficacy of nivolumab in the DG01 study.

### Statistical analyses

The sample size was set at 100 patients in view of the feasibility of data collection at the participating institutions. For OS, rwPFS, and other outcomes, the median survival time and the point estimates of survival (6, 12, 18, 24, and 36 months) were estimated using the Kaplan–Meier method for the entire cohort and for each treatment group. The Brookmeyer and Crowley method was used to calculate the 95% confidence interval (CI) for the median survival time, and the Greenwood formula was applied to calculate the 95% CI for the survival rate. Similar estimates of OS were obtained for subgroup analysis. ORR and DCR were estimated in patients with evaluable target lesions. Exploratory analysis for factors affecting OS was conducted using a multivariate Cox analysis model with HER2 status as a covariate with a two-sided significance level of 0.05. All analyses were performed using SAS version 9.4 (SAS Institute Inc., Cary, NC, USA). Confounders were not considered in the DG01 similarly matched analysis.

## Results

### Patient disposition, demographics, and baseline characteristics

**Fig. 1:**
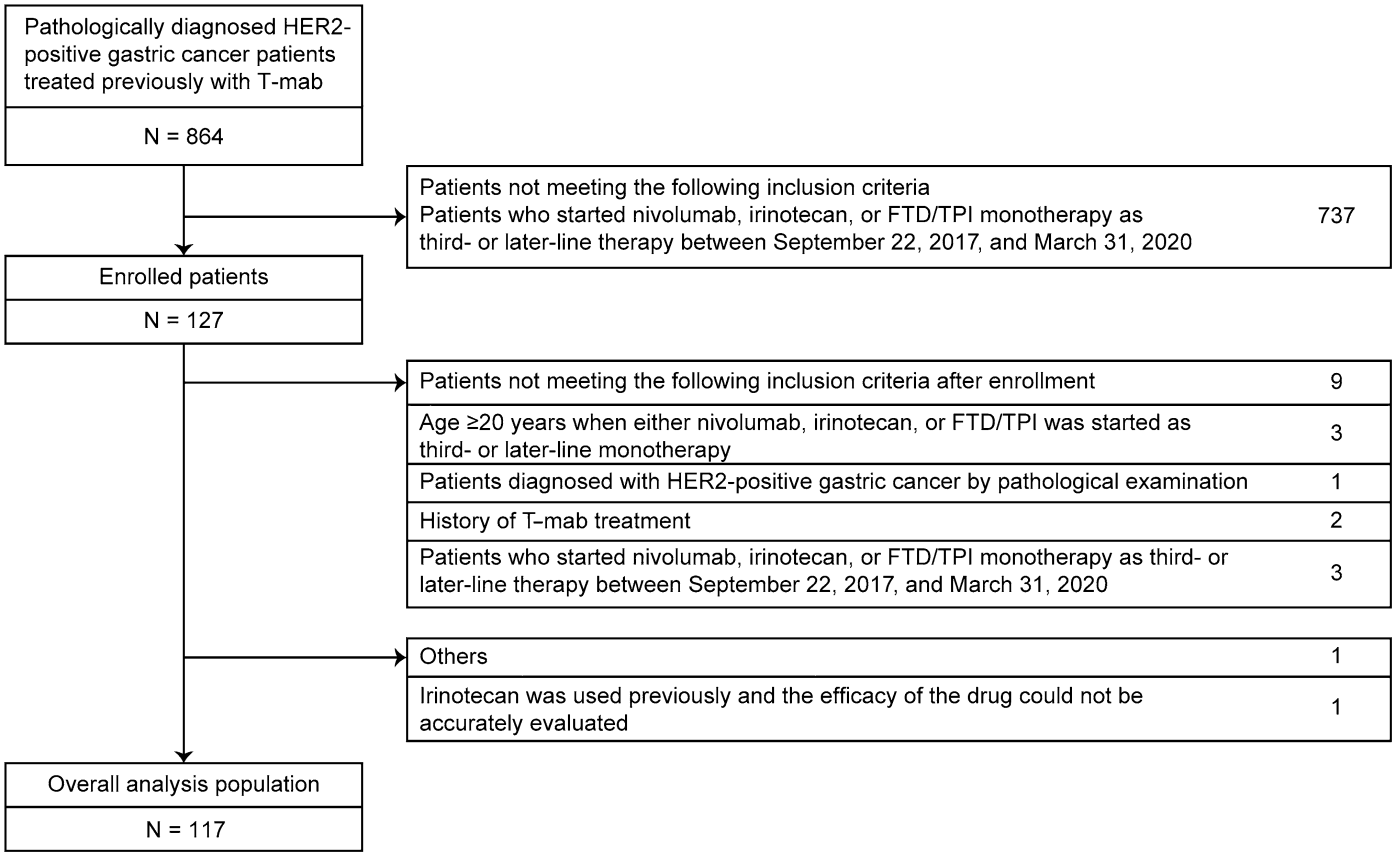
Patient disposition. *CI* confidence interval, FTD*/TPI* trifluridine/tipiracil, *HER2* human epidermal growth factor receptor 2, *T-mab* trastuzumab

A total of 864 patients with HER2-positive unresectable or recurrent gastric cancer who were previously treated with trastuzumab were identified, of whom 127 patients were enrolled. Among them, 117 patients met the eligibility criteria and constituted the overall analysis population (Fig. 1).

The median (range) age of the overall analysis population was 71 (38–89) years, and 84.6% of patients had an ECOG PS of 0–1. The most commonly prescribed 3L + monotherapy was nivolumab (*n* = 100), followed by irinotecan (*n* = 12) and FTD/TPI (*n* = 5). The median (range) number of previous lines of treatment was 2 (2–6), and the median (range) duration from first-line treatment to initiation of third- or later-line monotherapy was 431 (112–1903) days (Table 1). The median (range) follow-up period was 150 (25–1007) days.

**Table 1:** Patient demographics and baseline characteristics

Parameter	Overall (*N* = 117)	Nivolumab (*N* = 100)	Irinotecan (*N* = 12)	FTD/TPI (*N* = 5)
Age (years)				
All, median (range)	71 (38–89)	71 (38–89)	71 (53–78)	74 (69–78)
Sex				
Male	92 (78.6)	78 (78.0)	9 (75.0)	5 (100.0)
Surgery for the primary lesion				
Yes	63 (53.8)	54 (54.0)	6 (50.0)	3 (60.0)
No	54 (46.2)	46 (46.0)	6 (50.0)	2 (40.0)
Histology of the primary lesion				
Diffuse type	26 (22.2)	24 (24.0)	1 (8.3)	1 (20.0)
Intestinal type	84 (71.8)	70 (70.0)	11 (91.7)	3 (60.0)
Other	7 (6.0)	6 (6.0)	0	1 (20.0)
Primary tumor site				
Stomach	99 (84.6)	86 (86.0)	8 (66.7)	5 (100.0)
GE junction	18 (15.4)	14 (14.0)	4 (33.3)	0
HER2 status at initial treatment				
Positive	117 (100.0)	100 (100.0)	12 (100.0)	5 (100.0)
IHC3 +	83 (70.9)	71 (71.0)	10 (83.3)	2 (40.0)
IHC2 + and ISH +	33 (28.2)	28 (28.0)	2 (16.7)	3 (60.0)
Unknown^a^	1 (0.9)	1 (1.0)	0	0
Number of prior treatment lines				
All, median (range)	2 (2–6)	2 (2–6)	2 (2–3)	2 (2–3)
2	71 (60.7)	58 (58.0)	9 (75.0)	4 (80.0)
3	32 (27.4)	28 (28.0)	3 (25.0)	1 (20.0)
≥ 4	14 (12.0)	14 (14.0)	0	0
Prior treatment				
Trastuzumab	117 (100.0)	100 (100.0)	12 (100.0)	5 (100.0)
Ramucirumab	94 (80.3)	85 (85.0)	5 (41.7)	4 (80.0)
Taxane	109 (93.2)	93 (93.0)	11 (91.7)	5 (100.0)
Platinum	106 (90.6)	90 (90.0)	11 (91.7)	5 (100.0)
Pyrimidine fluoride	116 (99.1)	99 (99.0)	12 (100.0)	5 (100.0)
Irinotecan	16 (13.7)	16 (16.0)	0	0
Immune checkpoint inhibitor	1 (0.9)^b^	0	1 (8.3)^b^	0
Others	21 (17.9)	19 (19.0)	2 (16.7)	0
ECOG PS at the beginning of the current treatment				
0	35 (29.9)	31 (31.0)	4 (33.3)	0
1	64 (54.7)	53 (53.0)	6 (50.0)	5 (100.0)
2 or more	16 (13.7)	14 (14.0)	2 (16.7)	0
Unknown	2 (1.7)	2 (2.0)	0	0
Site of metastasis at the start of the current treatment				
Lymph nodes	66 (56.4)	54 (54.0)	10 (83.3)	2 (40.0)
Liver	58 (49.6)	52 (52.0)	3 (25.0)	3 (60.0)
Peritoneum	36 (30.8)	29 (29.0)	4 (33.3)	3 (60.0)
Lungs	21 (17.9)	17 (17.0)	4 (33.3)	0
Bone	4 (3.4)	3 (3.0)	1 (8.3)	0
Brain	1 (0.9)	1 (1.0)	0	0
Other	15 (12.8)	13 (13.0)	2 (16.7)	0
Time from initiation of first-line treatment to initiation of the current treatment (days)				
Median (interquartile range)	431 (304–752)	430 (304–748)	677 (379–890.5)	304 (278–357)
Min, max	112, 1903	112, 1903	133, 1596	248, 519

Data are *n* (%) unless specified otherwise

*ECOG PS* Eastern Cooperative Oncology Group performance status, *FTD/TPI* trifluridine/tipiracil, *GE* gastroesophageal, *HER2* human epidermal growth factor receptor 2, *IHC* immunohistochemistry, *ISH* in situ hybridization, *max* maximum, *min* minimum

^a^Although this patient’s HER2 was positive, the HER2 status could not be confirmed

^b^Pembrolizumab

### Exposure and subsequent treatment

The median (range) duration of treatment was 56 (2–800), 82 (28–155), and 176 (29–220) days in the nivolumab, irinotecan, and FTD/TPI treatment groups, respectively. At data cutoff, study treatment was permanently discontinued in 97.0% (97/100), 100% (12/12), and 100% (5/5) of patients in the nivolumab, irinotecan, and FTD/TPI treatment groups, respectively. The reasons for treatment discontinuation included PD (85.6%, 91.7%, and 80.0%, respectively) or others.

Of 117 eligible patients, 109 (93.2%) received a subsequent treatment regimen (Online Resource Table 1).

### Effectiveness

**Fig. 2:**
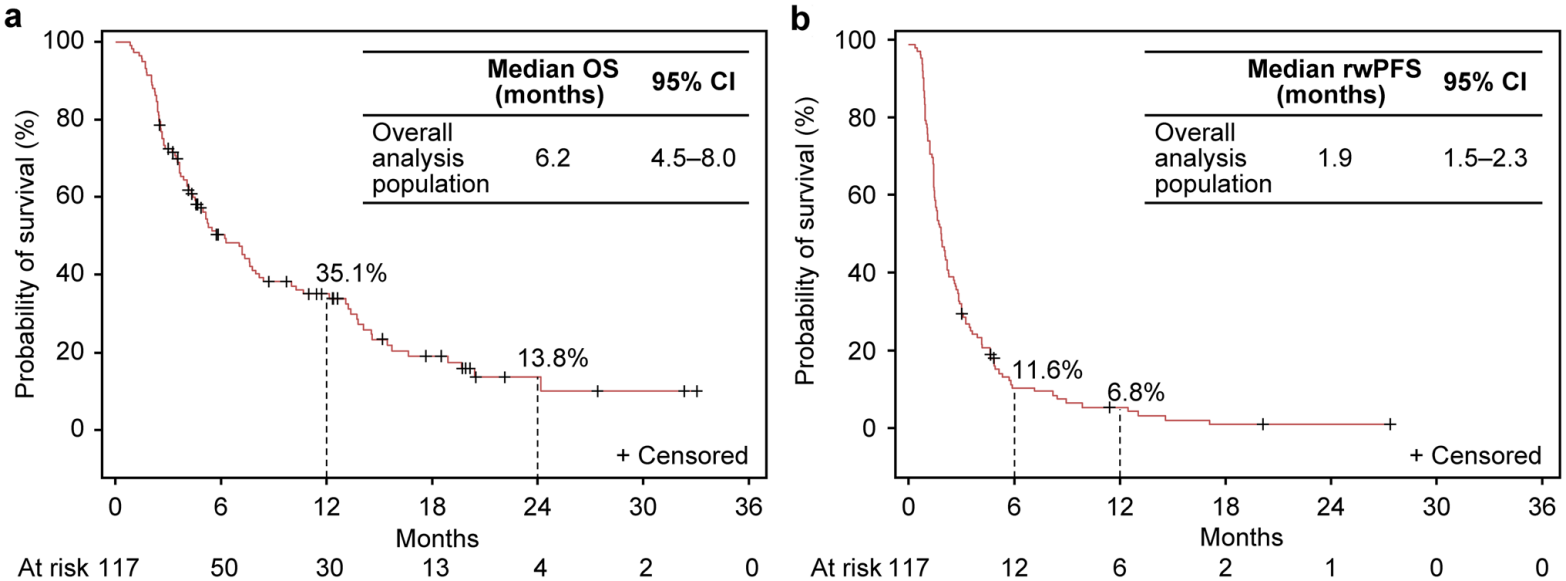
Kaplan–Meier plots of **a** OS and **b** rwPFS in the overall population. *CI* confidence interval, *OS* overall survival, *rwPFS* real-world progression-free survival

By Kaplan–Meier analysis, the median (95% CI) OS (Fig. 2a), rwPFS (Fig. 2b), and TTF (Online Resource Fig. 1a) in the overall analysis population were 6.2 (4.5–8.0), 1.9 (1.5–2.3), and 1.8 (1.5–2.2) months, respectively.

The overall survival rate % (95% CI) was 50.4 (40.8–59.3), 35.1 (26.1–44.3), 19.4 (11.8–28.3), and 13.8 (7–23) at the 6-, 12-, 18-, and 24-month follow-ups, respectively, in the overall analysis population. When stratified by treatment group, the median OS (95% CI) in the nivolumab, irinotecan, and FTD/TPI treatment groups was 5.3 (4.0–7.6), 9.5 (2.5–24.2), and 7.8 (2.6–7.8) months, respectively, and the survival rates also followed a similar trend (Online Resource Fig. 2). The median (95% CI) rwPFS in the nivolumab, irinotecan, and FTD/TPI treatment groups was 1.8 (1.5–2.2), 2.7 (0.9–4.2), and 5.8 (1.0–5.9) months, respectively. The median (95% CI) TTF in the nivolumab, irinotecan, and FTD/TPI treatment groups was 1.7 (1.4–2.1), 2.7 (0.9–4.2), and 5.8 (1.0–7.2) months, respectively. The ORR (CR + PR) and DCR for the 100 patients with evaluable target lesions were 9.0% (1% + 8%) and 32.0%, respectively. The ORR and DCR for the 87 patients in the nivolumab group were 9.2% (1.1% + 8.0%) and 27.6%, respectively (Table 2). The median (95% CI) DOR in the overall analysis population was 8.4 (4.2–12.9) months (Online Resource Fig. 1b).

**Table 2:** ORR and DCR in patients with evaluable target lesions

Category	Patients with measurable lesions			
	Overall (*N* = 100)	Nivolumab (*N* = 87)	Irinotecan (*N* = 9)	FTD/TPI (*N* = 4)
ORR, % (95% CI^a^)	9.0 (0.04–0.16)	9.2 (0.04–0.17)	11.1 (0.003–0.48)	0 (0.00–0.60)
CR	1 (1.0)	1 (1.1)	0	0
PR	8 (8.0)	7 (8.0)	1 (11.1)	0
SD	23 (23.0)	16 (18.4)	5 (55.6)	2 (50.0)
PD	67 (67.0)	62 (71.3)	3 (33.3)	2 (50.0)
NE	1 (1.0)	1 (1.1)	0	0
DCR, % (95% CI^a^)	32.0 (0.23–0.42)	27.6 (0.19–0.38)	66.7 (0.30–0.93)	50.0 (0.07–0.93)

Data are *n* (%) unless specified otherwise

*CI* confidence interval, *CR* complete response, *DCR* disease control rate, *FTD/TPI* trifluridine/tipiracil, *NE* not evaluable, *ORR* objective response rate, *PD* progressive disease, *PR* partial response, *SD* stable disease

^a^Clopper–Pearson exact 95% CI

In the DG01 similarly matched analysis, the median (95% CI) OS was 8.4 (5.1–13.4) months for the matched overall analysis population (*n* = 84) and 7.7 (4.7–13.2) months for the matched nivolumab group (*n* = 72; Table 3).

**Table 3:** Exploratory analysis of effectiveness in the DESTINY-Gastric01 (DG01) similarly matched population

	DESTINY-Gastric01 similarly matched population^a^			
	Median OS (95% CI), months	Median rwPFS (95% CI), months	Median TTF (95% CI), months	ORR (CR + PR) %
Overall (*n* = 84)	8.4 (5.1–13.4)	2.1 (1.6–2.9)	2.0 (1.5–2.8)	10.7 (1.2 + 9.5)
Nivolumab (*n* = 72)	7.7 (4.7–13.2)	1.9 (1.5–2.7)	1.9 (1.5–2.3)	11.1 (1.4 + 9.7)

*CI* confidence interval, *CR* complete response, *ECOG PS* Eastern Cooperative Oncology Group performance status, *ORR* objective response rate, *OS* overall survival, *PR* partial response, *rwPFS* real-world progression-free survival, *TTF* time to treatment failure

^a^The overall population and nivolumab subgroup in this study matched to the DESTINY-Gastric01 partial inclusion criteria, ECOG PS (0–1) with evaluable lesions

Multivariate Cox regression analysis showed that men vs women, presence vs absence of a primary lesion, diffuse type vs intestinal type, median neutrophil–lymphocyte ratio ≥ 2.54 vs < 2.54, Glasgow prognostic score 1–2 vs 0 and 2 vs 0–1, presence vs absence of hepatic metastasis, presence vs absence of peritoneal dissemination, and ECOG PS ≥ 1 vs 0 and ≥ 2 vs 0–1 were factors associated with OS in the overall analysis population (Table 4).

**Table 4:** Analysis of factors influencing OS using the multivariate Cox analysis model

Item	Category	Overall (*N* = 117)			Nivolumab (*N* = 100)		
		Adjusted HR^a^	95% CI	*p* value^b^	Adjusted HR^a^	95% CI	*p* value^b^
Age	≥ 65 years vs < 65 years	0.69	0.42–1.12	0.13	0.56	0.33–0.94	0.03
	≥ 75 years vs < 75 years	0.97	0.62–1.53	0.90	0.80	0.49–1.31	0.37
Sex	Men vs women	0.54	0.33–0.89	0.02	0.62	0.36–1.06	0.08
Treatment line	Fourth line vs third line	1.05	0.57–1.93	0.88	0.99	0.53–1.84	0.98
Presence or absence of a primary lesion	Yes vs no	0.56	0.37–0.86	0.008	0.60	0.38–0.95	0.03
Primary site	Gastric vs GE junction	1.62	0.86–3.05	0.14	1.18	0.59–2.37	0.65
Histological type	Diffuse type vs intestinal type	2.20	1.34–3.62	0.002	2.09	1.24–3.49	0.005
NLR^c^	≥ 2.54^f^ vs < 2.54^f^	2.29	1.49–3.52	< 0.001	2.30	1.45–3.66	< 0.001
LMR^d^	≥ 3.00^f^ vs < 3.00^f^	0.86	0.56–1.31	0.48	0.84	0.53–1.33	0.46
GPS^e^	1–2 vs 0	2.13	1.38–3.28	0.001	2.49	1.54–4.02	< 0.001
	2 vs 0–1	2.31	1.41–3.78	0.001	2.19	1.30–3.67	0.003
LDH (U/L)	≥ 222 vs < 222	1.40	0.92–2.15	0.12	1.37	0.87–2.16	0.17
ALP (U/L)	≥ 322 vs < 322	1.36	0.89–2.08	0.16	1.42	0.89–2.26	0.14
Number of metastatic organs	≥ 2 vs 01	1.44	0.94–2.22	0.09	1.42	0.90–2.25	0.14
Hepatic metastasis	Yes vs no	1.92	1.25–2.95	0.003	1.82	1.15–2.90	0.01
Peritoneal dissemination	Yes vs no	1.69	1.07–2.67	0.02	1.44	0.88–2.37	0.15
Measurable lesions	Yes vs no	0.73	0.40–1.31	0.29	0.86	0.44–1.68	0.66
ECOG PS	≥ 1 vs 0	1.90	1.16–3.10	0.01	2.05	1.20–3.48	0.008
	≥ 2 vs 0–‍1	3.71	2.05–6.73	< 0.001	3.50	1.85–6.63	< 0.001

*ALP* alkaline phosphatase, *CI* confidence interval, *ECOG PS* Eastern Cooperative Oncology Group performance status, *GE* gastroesophageal*, GPS* Glasgow Prognostic Score, *HER2* human epidermal growth factor receptor 2, *HR* hazard ratio, *LDH* lactate dehydrogenase, *LMR* lymphocyte-monocyte ratio, *NLR* neutrophil–lymphocyte ratio, *OS* overall survival

^a^HRs for each item with HER2 status as a covariate (using the right side of the category as the reference stratum)

^b^*p* value using the Wald test

^c^NLR = (% neutrophils/% lymphocytes)

^d^LMR = (% lymphocytes/% monocytes)

^e^GPS, 0: CRP ≤ 1.0 mg/dL and albumin ≥ 3.5 g/dL; 1, CRP > 1.0 mg/dL or albumin < 3.5 g/dL; 2, CRP > 1.0 mg/dL and albumin < 3.5 g/dL

^f^Median

Similarly, factors associated with OS in the nivolumab group were age ≥ 65 vs < 65 years, presence vs absence of a primary lesion, diffuse type vs intestinal type, median neutrophil–lymphocyte ratio ≥ 2.54 vs < 2.54, Glasgow prognostic score 1–2 vs 0 and 2 vs 0–1, presence vs absence of hepatic metastasis, and ECOG PS ≥ 1 vs 0 and ≥ 2 vs 0–1 (Table 4). Overall, statistical comparison across the groups was not feasible because of the small number of patients in the irinotecan and FTD/TPI groups.

## Discussion

This retrospective, observational study assessed physicians’ preferences and the effectiveness of third- or later-line monotherapy for HER2-positive advanced G/GEJ cancer in a daily clinical setting in Japan. This study included patients who had been previously treated with trastuzumab to identify HER2-positive patients before histopathological confirmation. The results indicate that nivolumab was most commonly prescribed as a third- or later-line treatment among the three standard-of-care monotherapies used in 100 of 117 patients analyzed in a real-world clinical scenario in Japan.

The exploratory subgroup analysis of nivolumab-treated patients with prior trastuzumab use in the ATTRACTION-2 trial concluded that nivolumab was efficacious and safe as a third- or later-line therapy regardless of prior trastuzumab use in patients with advanced G/GEJ cancer [17]. A longer median OS (8.3 months) [17] was observed in the ATTRACTION-2 subgroup analysis compared with the nivolumab group (5.3 months) in this study. On the other hand, the median PFS in nivolumab-treated patients with prior trastuzumab use (1.6 months) in the ATTRACTION-2 trial [17] was similar to the rwPFS in the nivolumab group (1.8 months) in this study. This study included patients with poor prognostic factors, such as poor ECOG PS, and the median OS was not better than those observed in previous randomized clinical trials [16, 17]. Taken together, the real-world therapeutic effectiveness of nivolumab, irinotecan, and FTD/TPI as third- or later-line treatment for HER2-positive, advanced gastric cancer may be limited.

It was reported that immune checkpoint inhibitors are unlikely to be effective in patients with rapidly progressive gastric cancer [10, 18]. Accelerated tumor growth or hyperprogressive disease (HPD) has been reported to occur in more than 21.0% (range: 21.0–29.4%) of patients with advanced gastric cancer during treatment with nivolumab and 13.5% of patients during treatment with irinotecan [19, 20], and poor ECOG PS, liver metastases, and a large sum of target lesion diameters have been identified as risk factors for HPD [19]. The OS subgroup analysis of the current study did identify presence vs absence of hepatic metastasis, presence vs absence of peritoneal dissemination (except nivolumab-treated group), and ECOG PS ≥ 1 vs 0 and ≥ 2 vs 0–1 status, among others, as risk factors that impaired OS benefit in both the overall analysis population and the nivolumab-treated group. The prognostic factors that were identified are consistent with the previous findings regarding factors that were associated with a decrease in PFS in patients treated with third-line nivolumab vs irinotecan for advanced gastric cancer [21] and may, therefore, have contributed to the limited OS benefit of nivolumab observed in the current study.

Although the irinotecan group had a small sample size of 12 patients, the treatment outcome in terms of effectiveness (ORR: 11.1%; DCR: 66.7%; median OS: 9.5 months; and median rwPFS: 2.7 months) was similar to that with DG01 PC of chemotherapy (ORR: 14.0%; DCR: 62.0%; median OS: 8.4 months; and PFS: 3.5 months) [16].

In general, nivolumab, irinotecan, and FTD/TPI are not HER2-targeted therapies but are effective irrespective of HER2 status or prior trastuzumab use [11–13, 16, 17]. In a subgroup analysis of the phase 3 ATTRACTION-2 trial, Satoh et al. demonstrated that nivolumab showed significant differences in median OS with prior trastuzumab use versus without prior trastuzumab use (8.3 vs 4.8 months; *p* = 0.04). However, it was not concluded that prior trastuzumab use affects OS, as there were no significant differences in PFS between the two groups. Moreover, differences in patient backgrounds and numbers of post-progression pharmacotherapies were also noted [17]. Furthermore, there might be a relationship between HER2 status and immune checkpoint inhibitors; however, the mechanism, such as the relationship between HER2 status and PD-L1 expression, is still unclear [17, 21]. Based on the above information, the association between HER2 status and nivolumab efficacy is unclear. In a salvage-line setting after 3rd-line treatment, in addition to the level of pretreatment, adverse prognostic factors such as PS and hepatic metastatic disease may affect salvage-line treatment, limiting the efficacy of drugs that are not anti-HER2 therapies.

T-DXd has shown promising results in clinical trials (ORR: 51%; DCR: 86%; median OS: 12.5 months; and median PFS: 5.6 months) and was approved in Japan in September 2020 for the treatment of patients with HER2-positive unresectable or metastatic G/GEJ cancer that progressed on cancer chemotherapy [16]. It is currently recommended as a third-line treatment option for patients with HER2-positive gastric cancer per the Japanese guidelines [16, 22–24]. T-DXd has the potential to significantly change the real-world use of third- or later-line treatment in Japan. In addition, the HER2-positive gastric cancer treatment paradigm in the US may also change because T-DXd is approved as second- or later-line therapy.

In the DG01 similarly matched population analysis, it is speculated that this result may demonstrate the efficacy of nivolumab in DG01, and indirectly, the clinical utility of T-DXd. In the future, we believe that optimal treatment selection should be pursued through collection of real-world effectiveness and safety data, including those for T-DXd.

Study limitations included the fact that the differences in patient numbers did not allow for definitive comparisons between nivolumab, irinotecan, or FTD/TPI in terms of the optimal treatment choice. The data evaluated in this study were collected before the approval of T-DXd so there were no real-world data on T-DXd in this study. Lesion assessment and timing of imaging were not defined owing to the retrospective nature of the study, and no central review was performed for tumor response evaluation. The DG01 similarly matched analysis was conducted in this study on patients matched to ECOG PS (0–1) with evaluable lesions only. Furthermore, only real-world effectiveness was evaluated, and safety was not investigated. Despite these limitations, the results of the current study are highly generalizable to real-world situations among Japanese patients with HER2-positive, advanced gastric cancer at the time of the research because it employed a retrospective chart review design with 20 contributing facilities.

## Conclusion

The results of this study revealed that nivolumab was the mainstay of treatment in Japanese patients with HER2-positive, advanced gastric cancer during the study period. The observed OS in this study was shorter than that in clinical trials, suggesting that the real-world effectiveness of 3L + therapies may be limited. Evaluation of a new HER2 agent, T-DXd, is warranted for potential improvement in the real-world outcome and prognosis of patients with HER2-positive gastric cancer.

## Supplementary Information

Below is the link to the electronic supplementary material.

Supplementary file1 (DOCX 586 kb)
