# Supplementary material for: Real-world effectiveness of third- or later-line treatment in Japanese patients with HER2-positive, unresectable, recurrent or metastatic gastric cancer: a retrospective observational study
Source: Int J Clin Oncol. 2022 Apr 30;27(7):1154–63. doi: 10.1007/s10147-022-02162-4 (PMC9209345; doi:10.1007/s10147-022-02162-4)
Supplement: Supplementary file 1 — Supplementary file1 (DOCX 586 kb) [file 10147_2022_2162_MOESM1_ESM.docx]

**Electronic Supplementary Material**

**Title:** Real-world effectiveness of third- or later-line treatment in Japanese patients with HER2‑positive, unresectable, recurrent or metastatic gastric cancer: a retrospective observational study

**Target journal:** *International Journal of Clinical Oncology*

**Authors:**

Daisuke Sakai^1^, Takeshi Omori^2^, Soichi Fumita^3^, Junya Fujita^4^*, Ryohei Kawabata^5^**, Jin Matsuyama^6^, Hisateru Yasui^7^, Motohiro Hirao^8^, Tomono Kawase^9^, Kentaro Kishi^10^, Yoshiki Taniguchi^11^, Yasuhiro Miyazaki^12^, Junji Kawada^13^, Hironaga Satake^14^, Tomoko Miura^15^, Akimitsu Miyake^16^***, Yukinori Kurokawa^1^, Makoto Yamasaki^1^****, Tomomi Yamada^16^, Taroh Satoh^1^, Hidetoshi Eguchi^1^, Yuichiro Doki^1^

**Affiliations:**

^1^Osaka University Graduate School of Medicine, Suita, Japan

^2^Osaka International Cancer Institute, Osaka, Japan

^3^Kindai University, Osakasayama, Japan

^4^Sakai City Medical Center, Sakai, Japan

^5^Osaka Rosai Hospital, Sakai, Japan

^6^Higashiosaka City Medical Center, Higashiosaka, Japan

^7^Kobe City Medical Center General Hospital, Kobe, Japan

^8^National Hospital Organization Osaka National Hospital, Osaka, Japan

^9^Toyonaka Municipal Hospital, Toyonaka, Japan

^10^Osaka Police Hospital, Osaka, Japan

^11^Saiseikai Senri Hospital, Suita, Japan

^12^Osaka General Medical Center, Osaka, Japan

^13^Yao Municipal Hospital, Yao, Japan

^14^Kansai Medical University, Hirakata, Japan

^15^Daiichi Sankyo Co., Ltd., Tokyo, Japan

^16^Osaka University Hospital, Suita, Japan

*Current affiliation: Yao Municipal Hospital, Yao, Japan

**Current affiliation: Sakai City Medical Center, Sakai, Japan

***Current affiliation: Tohoku University School of Medicine, Sendai, Japan

****Current affiliation: Kansai Medical University, Hirakata, Japan

**Corresponding author:**

Email: [dsakai@cfs.med.osaka-u.ac.jp](mailto:dsakai@cfs.med.osaka-u.ac.jp)

**Online Resource Fig. 1** Kaplan–Meier analysis of a) TTF and b) DOR

*CI* confidence interval, *DOR* duration of response, *TTF* time to treatment failure.


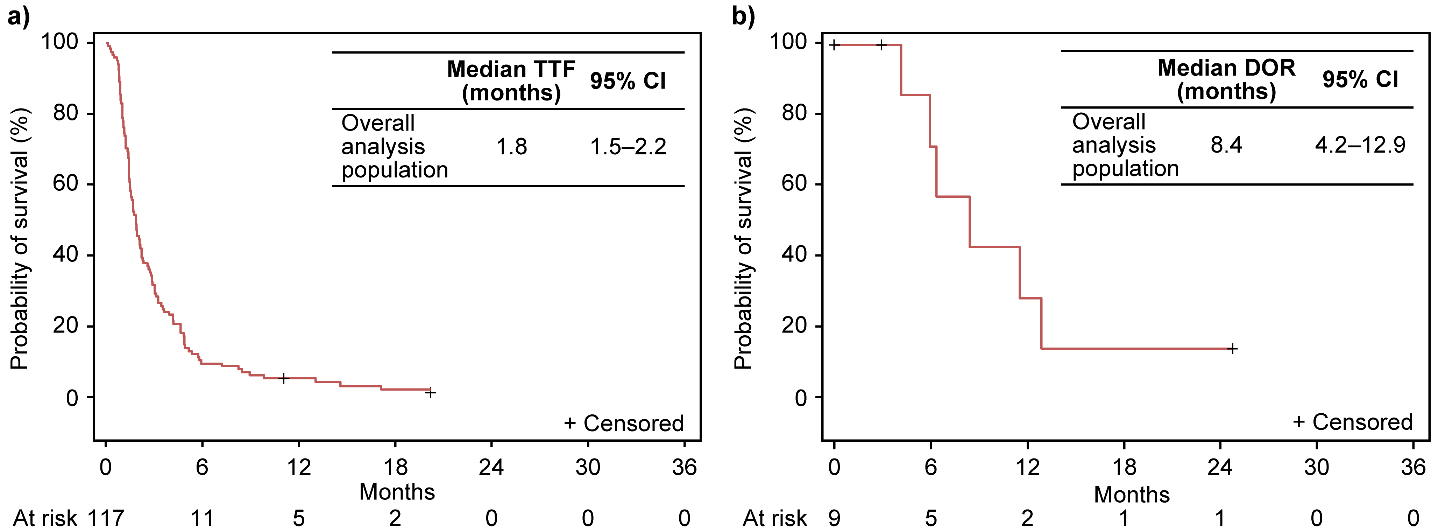


**Online Resource Fig. 2** Kaplan–Meier analysis of OS stratified by treatment

*CI* confidence interval, *FTD/TPI* trifluridine/tipiracil, *OS* overall survival.

**Online Resource Table 1** Summary of subsequent treatment

| Group, n (%) | | **All patients** | **Nivolumab** | **Irinotecan** | **FTD/TPI** |
| --- | --- | --- | --- | --- | --- |
| Total number of subsequent treatment | | 109 | 93 | 12 | 4 |
| Drug treatment | Irinotecan | 22 (20.2) | 22 (23.7) | 0 | 0 |
|  | Pyrimidine fluoride | 16 (14.7) | 11 (11.8) | 3 (25.0) | 2 (50.0) |
|  | Taxane | 13 (11.9) | 9 (9.7) | 4 (33.3) | 0 |
|  | Platinum | 12 (11.0) | 8 (8.6) | 3 (25.0) | 1 (25.0) |
|  | Immune checkpoint inhibitor^a^ | 11 (10.1) | 2 (2.2) | 7 (58.3) | 2 (50.0) |
|  | Ramucirumab | 11 (10.1) | 10 (10.8) | 1 (8.3) | 0 |
|  | Trastuzumab | 8 (7.3) | 7 (7.5) | 1 (8.3) | 0 |
|  | Others | 16 (14.7) | 13 (14.0) | 3 (25.0) | 0 |
| Radiation therapy | Yes | 4 (3.7) | 2 (2.2) | 2 (16.7) | 0 |
|  | No | 105 (96.3) | 91 (97.8) | 10 (83.3) | 4 (100.0) |
| Surgery | Yes | 0 | 0 | 0 | 0 |
|  | No | 109 (100.0) | 93 (100.0) | 12 (100.0) | 4 (100.0) |
| BSC | Yes | 65 (59.6) | 59 (63.4) | 5 (41.7) | 1 (25.0) |
|  | No | 44 (40.4) | 34 (36.6) | 7 (58.3) | 3 (75.0) |
| Other | Yes^b^ | 2 (1.8) | 2 (2.2) | 0 | 0 |
|  | No | 107 (98.2) | 91 (97.8) | 12 (100.0) | 4 (100.0) |

*BSC* best supportive care, *FTD/TPI* trifluridine/tipiracil.

^a^Nivolumab.

^b^Transcatheter arterial embolization, Denver shunt.

**Online Resource Table 2** List of participating facilities with names of the investigators

| **Name of the facility** | **Name of the investigator** |
| --- | --- |
| Osaka University Hospital | Daisuke Sakai |
| Osaka Rosai Hospital | Ryohei Kawabata |
| Osaka International Cancer Institute | Takeshi Omori |
| Sakai City Medical Center | Junya Fujita |
| Osaka General Medical Center | Kazumasa Fujitani |
| National Hospital Organization Osaka National Hospital | Motohiro Hirao |
| Yao Municipal Hospital | Junji Kawada |
| Saiseikai Senri Hospital | Hirokazu Taniguchi |
| Kansai Rosai Hospital | Atsushi Takeno |
| Toyonaka Municipal Hospital | Hiroshi Imamura |
| Higashiosaka City Medical Center | Jin Matsuyama |
| Minoh City Hospital | Takafumi Hirao |
| Rinku General Medical Center | Akina Furukawa |
| Osaka Police Hospital | Kentaro Kishi |
| Hyogo Prefectural Nishinomiya Hospital | Kazuyuki Okada |
| Otemae Hospital | Eiji Taniguchi |
| Ikeda City Hospital | Yusuke Akamaru |
| Kindai University Hospital | Hisato Kawakami |
| Kansai Medical University Hospital | Hironaga Satake |
| Kobe City Medical Center General Hospital | Hisateru Yasui |
